# Efficient silica synthesis from tetra(glycerol)orthosilicate with cathepsin- and silicatein-like proteins

**DOI:** 10.1038/s41598-018-34965-9

**Published:** 2018-11-13

**Authors:** Natalia V. Povarova, Nikolay A. Barinov, Mikhail S. Baranov, Nadezhda M. Markina, Anna M. Varizhuk, Galina E. Pozmogova, Dmitry V. Klinov, Valery B. Kozhemyako, Konstantin A. Lukyanov

**Affiliations:** 1 Shemyakin-Ovchinnikov Institute of Bioorganic Chemistry, Miklukho-Maklaya 16/10, 117997 Moscow, Russia; 2 Federal Research and Clinical Center of Physical-Chemical Medicine, Malaya Pirogovskaya 1a, 119435 Moscow, Russia; 3 Pirogov Russian National Research Medical University, Ostrovitianov 1, 117997 Moscow, Russia; 4 G.B. Elyakov Pacific Institute of Bioorganic Chemistry, Far Eastern Branch of the Russian Academy of Sciences, 690022 Vladivostok, Russia

## Abstract

Silicateins play a key role in biosynthesis of spicules in marine sponges; they are also capable to catalyze formation of amorphous silica *in vitro*. Silicateins are highly homologous to cathepsins L – a family of cysteine proteases. Molecular mechanisms of silicatein activity remain controversial. Here site-directed mutagenesis was used to clarify significance of selected residues in silica polymerization. A number of mutations were introduced into two sponge proteins – silicatein A1 and cathepsin L from *Latrunculia oparinae*, as well as into human cathepsin L. First direction was alanine scanning of the proposed catalytic residues. Also, reciprocal mutations were introduced at selected positions that differ between cathepsins L and silicateins. Surprisingly, all the wild type and mutant proteins were capable to catalyze amorphous silica formation with a water-soluble silica precursor tetra(glycerol)orthosilicate. Some mutants possessed several-fold enhanced silica-forming activity and can potentially be useful for nanomaterial synthesis applications. Our findings contradict to the previously suggested mechanisms of silicatein action via a catalytic triad analogous to that in cathepsins L. Instead, a surface-templated biosilification by silicateins and related proteins can be proposed.

## Introduction

Silicateins are the common spicule-forming proteins of marine sponges^1^. They are homologous to cathepsins – a family of proteases acting mainly in lysosomes at the low pH^2^. Most cathepsins are cysteine proteases with Cys-His-Asn catalytic triad, although some cathepsins are serine or aspartate proteases. Among different cathepsin groups, cathepsins L are the closest homologs of silicateins. In spite of the evolutionary and functional divergence, sponge silicateins share over 50% identity with human cathepsin L (CTSL). Amino acid alignment shows that the active site amino acids His and Asn are conserved, whereas position 26 (25 for CTSL) is occupied by Cys in CTSL and Ser in silicateins^1^. This Cys to Ser replacement is thought to be the key functional difference between the two protein classes. During spicule formation in sponges, silicateins interact with few more proteins, such as collagen, galectin, and silintaphins, and form long fibers, which promote silica polymerization around them^3^. Silicatein-associated proteins have an impact on the silicatein activity^4^, but silicatein itself is sufficient to form amorphous^5,6^ silica from different silica acid precursors *in vitro*.

**Figure 1:**
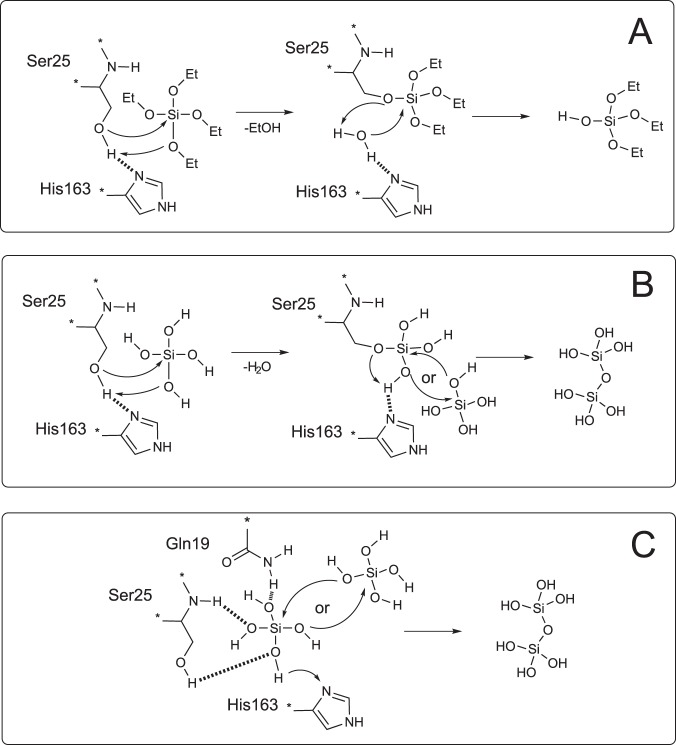
Previously proposed mechanisms of the silicatein activity. (**A**) Tetraethyl orthosilicate (TEOS) hydrolysis by silicatein^7^. (**B**,**C**) Two proposed mechanisms of silica acid condensation^8,9^.

Catalytic mechanism of the silicateins is not directly confirmed, but the key step is supposed to be a binding and activation of silica acid or silica acid precursor by the enzyme active site. Similarly to highly homologous CTSL, the active site is formed by Ser25 and His163. It is generally thought that these residues activate silicon atom of the substrate which results in hydrolysis (Fig. 1A)^7^ or polymerization (Fig. 1B)^8^, or both^9^. The exact molecular mechanism of interactions between the active site residues and substrate remains controversial. Some mechanisms suggest formation of the covalent bond between silicon atom and Ser26 (Fig. 1A,B)^9^, other include extra stabilization by Gln residue without covalent binding (Fig. 1C)^8^. Cysteine in the active site is supposed to be non-efficient for silica polymerization catalysis and is commonly described as a feature of cathepsins L^3^. Site-directed mutagenesis confirmed the crucial role of Ser26 and His165 in silicateins^7,10^.

However, two recent works demonstrated that in spite of the presence of Cys in the active site, cathepsin L can possess silica-polymerizing activity in some conditions^11,12^. We hypothesized that previously described effects of substitutions of the catalytic residues were overestimated due to the suboptimal silica acid precursor used. Recently, a highly water soluble silica acid precursor tetra(glycerol)orthosilicate (TGS) was introduced that provides a convenient and efficient way to assess protein activity^13^. Here, to clarify the role of certain amino acid residues, activity of silicatein and cathepsin L mutants with TGS was systematically evaluated.

## Results

### Protein isolation and purification

**Figure 2:**
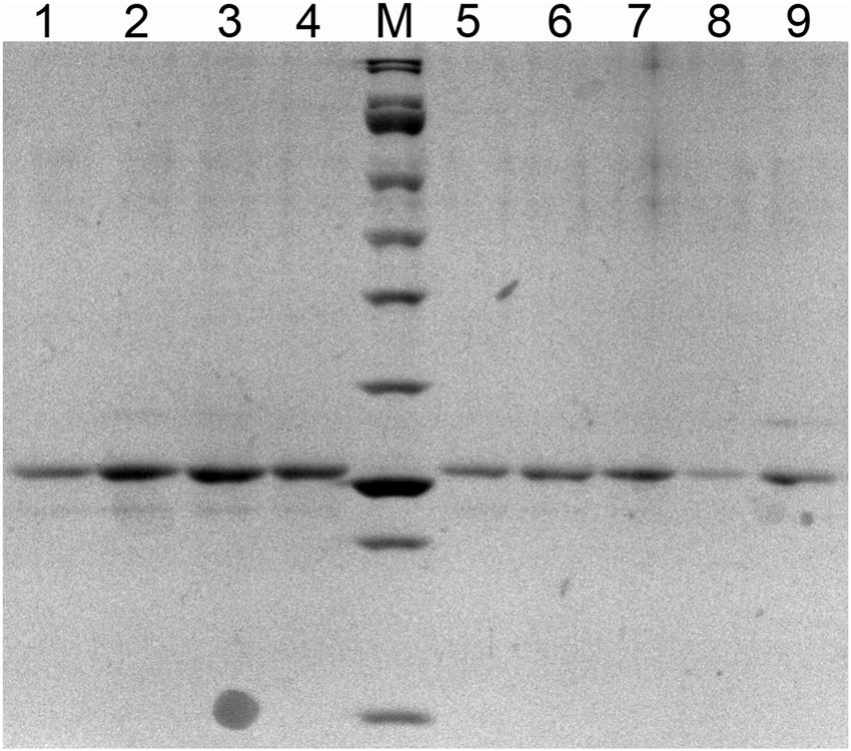
Gel-electrophoresis of sponge proteins LoSilA1, LoCath and mutants of LoCath. Lanes: 1 - LoSilA1, 2 - LoCath-N185A, 3 - LoCath-H165A, 4 - LoCath-Y27W, 5 - LoCath- K24G/S25A/C26S, 6 - LoCath-C26S, 7 - LoCath-C26A, 8 - LoCath-Q20A, 9 - LoCath. M - protein ladder (SibEnzyme), from bottom to top: 15, 20, 25, 30, 40, 50, 60, 85, 100, 150, 200 kDa.

Usually bacterially expressed silicateins are poorly soluble and require some extra tags to be produced in a soluble form^7^. Genes of two wild-type proteins from marine sponge *Latrunculia oparinae* were cloned, namely silicatein A1 (LoSilA1) and cathepsin L (LoCath), in several vectors, and different expression systems to express proteins were tested: pQE30 in XLBlue and BL21 strains, pBAD in BW and XJB strains, pET-40b(+) in XJB, BL and BL21codon+ strains. Also, different temperature (room temperature and 37 °C) and different concentration of inductors were tested. The conditions described in experimental section was the only one, which allowed to express proteins in soluble fraction. LoSilA1 and LoCath were still difficult to purify from the cell lysate by metal-affinity chromatography until His-tag was relocated to the C-terminus. The yield of the protein was about 1 mg per 1 liter of the cell culture. The purity of the proteins (more than 85%) was confirmed by the SDS-PAGE (representative example is shown in Fig. 2). Due to high protein similarity the same conditions were used for CTSL, but it was found that this protein should be expressed on 25 °C after induction.

**Figure 3:**
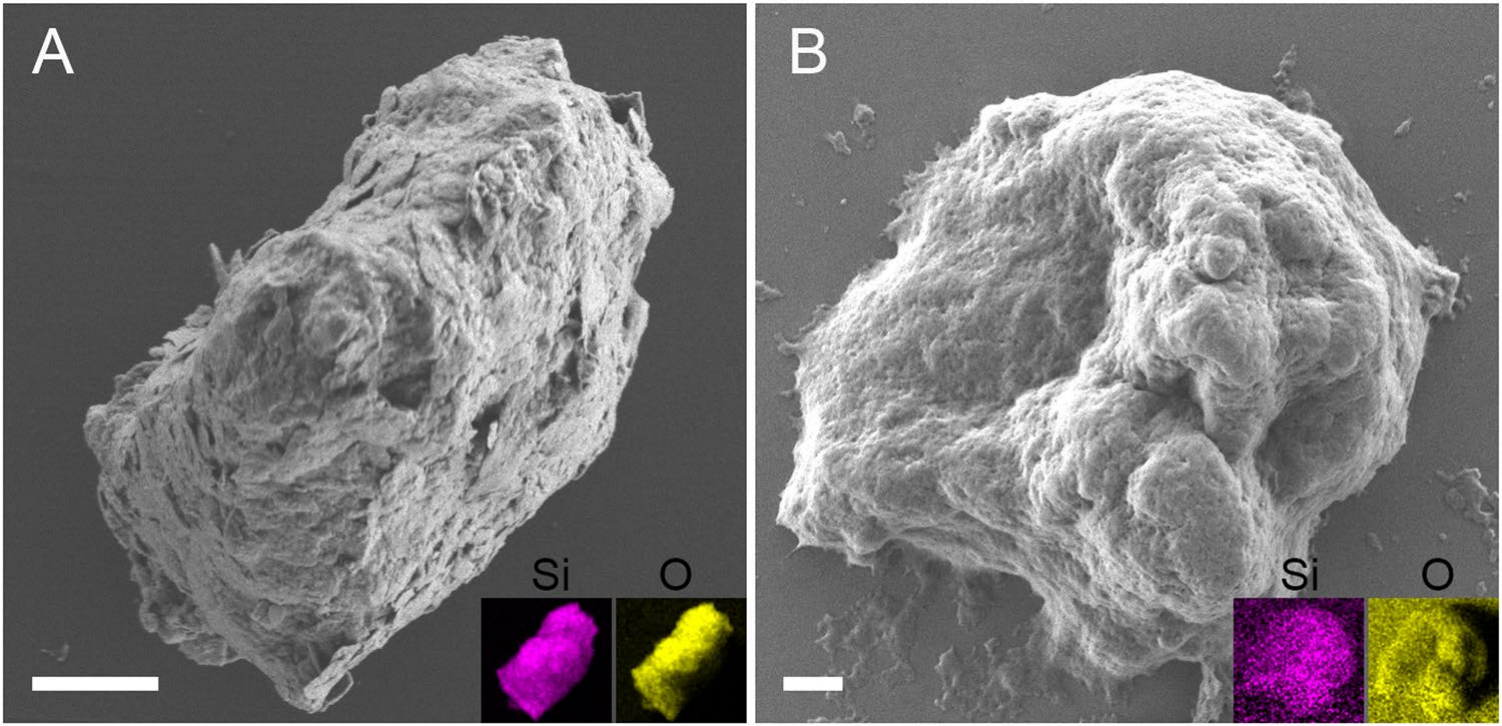
Scanning electron microscopy of silica particles. Shown are representative particles formed by LoSilA1 (**A**) and LoCath (**B**) with TGS. Scale bars 1 µm. Corresponding XRF maps of Si (magenta) and O (yellow) distribution are in the insets.

To verify protein functionality scanning electron microscopy (SEM) was used. The data showed that both LoSilA1 and LoCath form similar amorphous silica particles with TGS (Fig. 3).

### Mutagenesis of sponge *Latrunculia oparinae* proteins

**Figure 4:**
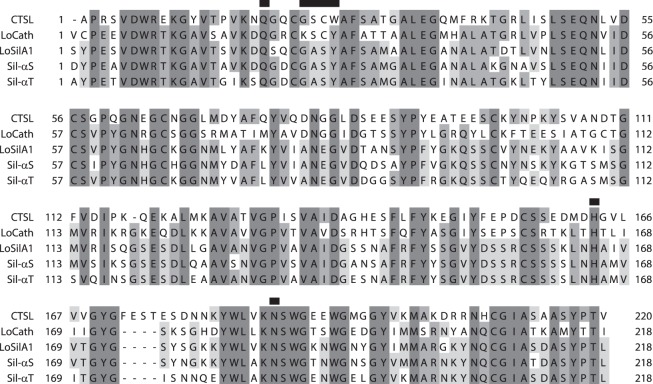
Protein sequence alignment. Shown are human cathepsin L (CTSL), *Latrunculia oparinae* silicatein A1 (LoSilA1), *L*. *oparinae* cathepsin (LoCath), *Tethya aurantium* silicatein-α (Sil-αT), and *Suberites domuncula* silicatein-α (Sil-αS). Mutated positions are marked with black bars. Numbering is based on LoSilA1, so the catalytic triad is Ser26, His165 and Asn185. For Cathepsin L catalytic triad is usually referred to as Ser25, His163 and Asn187.

Point mutations of the catalytic residues and their close neighbourhoods were introduced. Residues at positions 20, 26, 165 and 185 (here and below we use numbering according to mature form of the proteins, Fig. 4) were changed to Ala to test their proposed involvement into catalysis. Also, reciprocal mutations at the key catalytic position 26 – S26C in LoSilA1 and C26S in LoCath – were made. Notably, LoSilA1 and LoCath have very different residues upstream the catalytic Ser26 (or Cys26) – silicatein has GAS sequence at positions 24–26, while *L*. *oparinae* cathepsin L has KSC. So, triple mutants were made, where these moieties was reciprocally exchanged between the two proteins. In addition, Y27W substitution was introduced because this flanking residue is conservative in all the silica-condensing proteins, but differs in CTSL (Fig. 4).

In the previous work, pH optimum of LoSilA1 was measured to be 5.5^13^. LoCath has pI similar to that of LoSilA1, so both proteins were analyzed in the same conditions (PBS pH 5.5 at 0 °C). Kinetics of the silica formation was studied for LoSilA1 (0–120 min, with 20 min intervals). The reaction yield reached the maximum at 40 min (not shown). So, in the further experiments, 1-h reaction time was used to ensure completeness of the reaction together with low level of TGS spontaneous polymerization in this period of time^13^.

**Figure 5:**
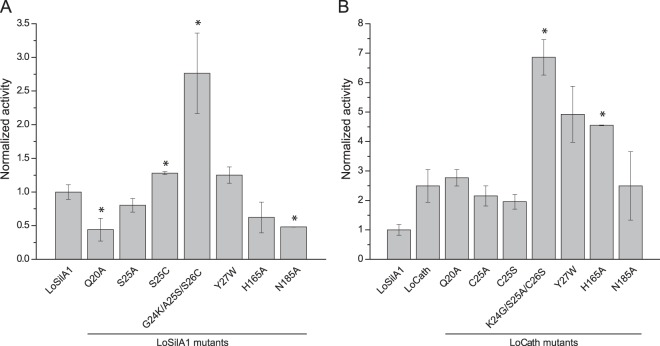
Silica-polymerizing activity of the *L*. *oparinae* proteins and their mutants. All values were normalized to the wild-type LoSilA1. (**A**) LoSilA1 mutants (absolute amount of the silica polymerized by LOSilA1 was 0.03 mM). (**B**) LoCath mutants (absolute amount of the silica polymerized by LoSilA1 was 0.06 mM). The data are represented by mean ± SD. The values were compared using two sample t-test (mutants vs the corresponding wild-type protein, wild-type LoCath was compared to wild-type LoSilA1), *corresponds to the p < 0.05.

Surprisingly, all the mutants demonstrated silica polymerizing activity, even proteins with alanine in the active site (Fig. 5). LoSilA1 Ala-mutants (Q20A, S26A, H165A, N185A) were slightly less active, while LoCath-H165A mutant was a bit more effective than the corresponding wild-type proteins. Substitutions Q20A, C26A, and N185A did not alter LoCath activity. LoSilA1-G24K/A25S/S26C mutant was almost 3-fold more efficient than the wild type LoSilA1. Cathepsin mutant LoCath-K24G/S25A/C26S demonstrated a similar value of activity enhancement compared to the parental protein.

None of the mutations abolished silica condensing activity of the proteins. These unexpected results turned our attention to CTSL. We hypothesized that CTSL could also demonstrate silica-polymerizing activity with TGS as a silica acid precursor.

### Analysis of human cathepsin L

SEM analysis of samples obtained by incubation of CTSL with TGS showed amorphous silica particles similar in shape and element composition to that formed by the sponge LoSilA1 and LoCath (Supplementary Fig. S1). At the same time, negative control samples of TGS with BSA or without any protein did not contain any silica particles (not shown).

Then influence of mutations on silica-polymerizing activity of CTSL was investigated. First, Ala-mutants of the catalytic triad were obtained. Further mutagenesis was based on the Fairhead *et al*. work. In this work authors detected silica polymerizing activity of silicatein-cathepsin L chimerae only after the replacement of the catalytic Cys and both flanking residues by the corresponding silicatein residues (SCW to ASY)^8^. Thus, three single-point mutants (G24A, C25S, W26Y), two double mutants (G24A/C25S and C25S/W26Y), and one triple mutant (G24A/C25S/W26Y) were obtained.

Analysis of silica polymerizing activity both in PBS pH 5.5 at 0 °C and Tris-HCl pH 6.8 at RT was performed, because neutral Tris-HCl was a common buffer for the silicatein analysis in other works^1,7^. BSA and fluorescent protein mKate with 6His-tag were used as negative controls.

**Figure 6:**
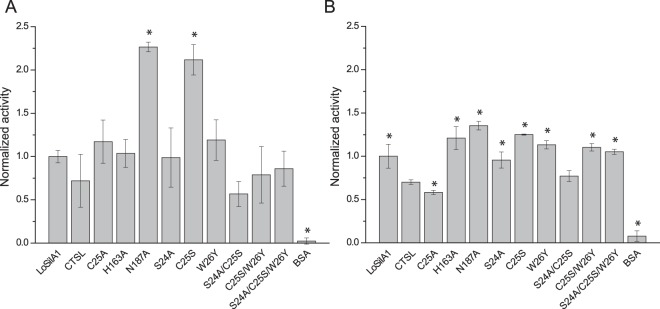
Silica polymerizing activity of wild-type human cathepsin L (CTSL) and its mutants. All values were normalized to the wild-type LoSilA1. Reactions were performed in PBS pH 5.5 at 0 °C (**A**) and Tris-HCl pH 6.8 at room temperature (**B**). The absolute amount of the silica polymerized by LoSilA1 was 0.2 mM and 0.1 mM in PBS and Tris-HCl, respectively. The data are represented by mean ± SD. The values were compared using two sample t-test (mutants vs the corresponding wild-type protein, wild-type CTSL was compared to wild-type LoSilA1), *corresponds to the p < 0.05.

CTSL and its mutants showed silica polymerizing activity comparable to that of sponge silicatein A1 (Fig. 6). In the acidic conditions (PBS pH 5.5, 0 °C), mutants C25S and N187A demonstrated about 3-fold enhanced activity compared to the parental CTSL, whereas other mutants showed no significant difference from CTSL. In neutral conditions (Tris-HCl pH 6.8, 22 °C) only C25A and G24A/C25S showed the activity similar to the wild-type CTSL, while other mutants were more active than the wild-type protein; again, C25S and N187A variants were the most active. BSA showed some low-level activity, 10- and 30-fold lower than LoSilA1 in Tris-HCl and PBS, respectively. Silica polymerizing activity of mKate-6His was below the detection limit.

### Secondary structure analysis

**Figure 7:**
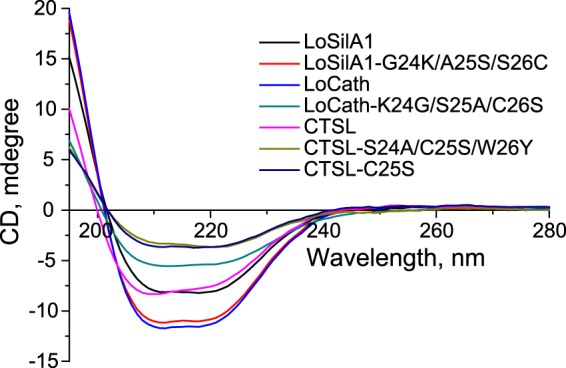
CD spectra of the proteins (the far UV region).

To analyze the effect of mutations on protein structure, the circular dichroism (CD) spectra were obtained for the wild-type proteins and some mutants at positions 23–26 (Fig. 7). CD spectra deconvolution showed that the wild-type proteins in the sodium-phosphate solution at pH 5.5 are well-folded and have a high content (>60%) of α-helix (Table 1). At the same time, three out of four studied mutants, namely LoCath-K24G/S25A/C26S, CTSL-G24A/C25S/W26Y, and CTSL-C25S showed strongly affected protein folding resulting in partial loss of α-helix elements (Table 1).

**Table 1:** CD-based analysis of the protein structures.

Secondary structure	Protein						
	LoSilA1	LoSilA1-G24K/A25S/S26C	LoCath	LoCath- K24G/S25A/C26S	CTSL	CTSL-G24A/C25S/W26Y	CTSL-C25S
α-helix (%)	77 ± 4	86 ± 2	87 ± 2	52 ± 3	63 ± 6	31 ± 1	33 ± 1
β-sheet (%)	3 ± 1	1 ± 1	1 ± 1	10 ± 1	8 ± 1	18 ± 1	18 ± 1
β-turn (%)	11 ± 1	8 ± 1	8 ± 1	14 ± 1	14 ± 1	17 ± 1	17 ± 1
Random coil (%)	9 ± 1	4 ± 1	4 ± 1	23 ± 1	16 ± 1	33 ± 1	32 ± 1

Percentages of the four main secondary structures were calculated by CDNN deconvolution analysis of the CD spectra with corresponding standard deviation error values.

## Discussion

In the present work we found that CTSL possesses silicatein-like activity and catalyzes silica polymerization. Analysis of the mutants of CTSL and *L*. *oparinae* LoSilA1 and LoCath showed that even mutants with the replacement of the one of the catalytic triad residues by alanine still have rather high silica-polymerizing activity. These observations contradict the generally accepted molecular mechanisms of silicatein enzymatic action^7–9,14^ and call for further investigation and interpretation.

Two reactions occur during silica condensation – hydrolysis of the silica acid precursor (Si-O bond hydrolysis) and condensation of the silica acid or partially hydrolyzed precursors (Si-O-Si bond formation)^15^. A protein can potentially catalyze: (i) hydrolysis, (ii) condensation, and (iii) both reactions.

It is difficult to discriminate between these possibilities as both hydrolysis and condensation can happen spontaneously, and thus one will observe protein-induced acceleration of formation of silica particles under any scenario. Nevertheless, there were several studies aiming to evaluate hydrolysis and condensation separately. Dakhili and coworkers studied silicatein α of *S*. *domuncula* with 4-nitrophenyl pivaloate or pivalamide as a substrate^10,16^. These compounds do not undergo further condensation, so, the experiment proved that silicatein is able to catalyze the hydrolysis reactions. Müller *et al*. demonstrated that the same silicatein could interact with bulky bis(p-aminophenoxy)-dimethylsilane which contains two Si-O and two S-C bonds, that confirms that silicatein could interact not only with orthosilicic acid itself^10,16^. Besides that, silicateins possess directly confirmed proteolytic activity, which is hydrolytic too^17–19^.

On the other hand, Fairhead *et al*. proved the silica condensing activity of the silicatein-cathepsin L chimera with sodium silicate. The hydrolysis step is absent is this system, the protein can catalyze only condensation reaction^8^.

Altogether these works demonstrated that the silicateins catalyze both the hydrolysis and condensation reactions. Mutations can affect both of them or one reaction only. In our experimental system, where TGS can be involved in every reaction, there is no possibility to distinguish effects. Due to spontaneous hydrolysis of TGS even mutants with no hydrolyzing activity could be defined as silica polymerizing.

Previous works on silicatein mutagenesis demonstrated a key role of Ser26 and His165 from the cathepsin-like catalytic triad, since S26A and H165A mutants failed to polymerize silica^7,10^. Both groups used tetraethyl orthosilicate (TEOS) as a silica acid precursor, which is poorly hydrolyzed in water at neutral pH. In contrast, here we used highly water soluble and hydrolysable TGS^13^ that can potentially unmask the remaining silica condensing activity of the mutants.

Fairhead *et al*. performed mutagenesis of CTSL to make a chimerae with the silica-condensing activity^8^. A variant similar to our CTSL-G24A/C25S/W26Y mutant (called AS2 in their work) was the first mutant with notable silica-condensing activity. In the present work, silica-forming activity somewhat increased in the row CTSL, CTSL-G24A/C25S/W26Y, CTSL-C25S, but all these proteins demonstrated comparable activities (see Fig. 6). Again, differences between results obtained in the present work and in the paper by Fairhead *et al*. can probably be attributed to different substrates used – TGS *versus* sodium silicate, respectively.

Another line of research suggested catalytic triad-independent mechanism of silicatein activity. For example, synthetic block copolypeptides with silica condensation activity were obtained^20^. All the lysine-containing polypeptides showed some activity; the most effective were poly-L-lysine-poly-L-cysteine block copolypeptides. Later, silica condensing activity was shown for cysteamine and even ethanolamine^21^. It should be noted that catalysis by the copolypeptides and small bifunctional molecules required much higher concentrations of silica precursor and catalyst, but the resulting particles of amorphous silica looked similar to that formed by the silicateins. Also, a surface-templated biosilification was proposed to occur in glass sponges with highly hydroxylated collagen^22^, histidine-rich protein glassin^23^ or even chitin^24–26^. The diversity of the involved polymers^27^ suggests some common principle of the biosilification instead of the unique enzyme mechanism for every compound. Indeed, collagen and chitin as regular polymers probably cannot form specific active sites for silica condensation. Moreover, silicateins are known to condense a variety of compounds – titanium dioxide, zirconia dioxide, poly(L-lactide), barium oxofluorotitanate, calcium carbonate, silver, and ceria oxide or ceria-zirconia oxide nanocrystals^28–34^. Silicatein is usually entrapped in the center of the formed particle. It can be hypothesized that silicatein can orient these substances onto its surface and ensure a templated growth of the particles^28^.

Our findings support the mechanism of surface-templated silica condensation by silicateins and related proteins. Silica acid precursor TGS has relatively high level of the spontaneous hydrolysis. Thus, even in the absence of hydrolytic activity the enzyme still has enough silica acid to work with, and alanine substitutions of the catalytic triad could be not crucial. At the same time, mutations affect the charge of the protein surface, protein conformation and possibly oligomerization that can result in the increased silica polymerization.

Importantly, it was demonstrated that silicatein-cathepsin L chimera loses its α-helix regions during the silica condensation^35^. In agreement with this work, there was a strong decrease in α-helix content in CD spectra of LoCath and CTSL mutants. Notably, the most crucial changes in the silica-condensing activity in our work were associated with substitutions at positions 24–26 in α-helix (Supplementary Fig. S2). Our data suggest that conformation of the protein affects their silica condensing or hydrolytic activity more than presence or absence of residues supposed to be catalytic.

A quite unexpected practical outcome of the present work was some mutants with enhanced silica-forming activity. In particular, sponge cathepsin L mutant LoCath-K24G/S25A/C26S possessed about 7-fold higher activity compared to the natural silicatein A1. Silicatein-directed formation of silica and other substances is considered to be a perspective way to nanomaterial synthesis with desired properties^32,36–38^. Cathepsin L mutants generated here or developed in further works might be a useful addition to the available silicateins. Finally, our data raise a fundamental question of whether cathepsins L and silicateins belong to clearly different functional classes as it is currently thought.

## Experimental procedures

### Cloning and protein Purification

Mutations were introduced by the AQUA cloning technique^39^ and confirmed by sequencing. Protein purification procedure was similar to described previously^13^. Genes were cloned into pET-40b(+) vector with C-terminal His-tag and without N-terminal tags (N-terminal DsbC- and His-tags were separated from the target proteins by two stop codons and a frame shift between them). Constructions were expressed in *E*. *coli* BL21-Codon+ strain. Expression was induced by 0.1 mM IPTG at 37 °C for 16 h for sponge proteins and at 25 °C for human cathepsin L. Cells were centrifuged, sonicated in 25 mM Tris-HCl pH 6.8, 150 mM NaCl with PMSF protease inhibitor (Thermo Fisher Scientific) and 5 mM DTT. Proteins were purified from the soluble fraction using Excel metal affinity resin (GE Healthcare Life Sciences), eluted with 25 mM Tris-HCl pH 6.8, 5 mM DTT, 200 mM imidazole, and stored at 4 °C for up to 3 days. Prior to the analysis, the proteins were transferred to assay buffer (25 mM Tris-HCl pH 6.8, 150 mM NaCl or PBS pH 5.5) using centrifugal filter units (Amicon ultra-4). Protein concentration was determined using optical density at 280 nm. Wild-type proteins, their mutants and reference silicatein A1 were isolated in parallel for each experiment. Purified protein samples were analysed using standard SDS-PAGE in 12% polyacrylamide gel stained with Coomassie Brilliant Blue G-250.

### CD spectra measurements

The circular dichroism (СD) spectra of the proteins (0.08 mg/ml in 0.06 M sodium phosphate buffer pH 5.5) were recorded using a Chirascan spectrophotometer (Applied Photophysics, UK), equipped with a thermostatic cuvette holder, in a 0.05-cm path length quartz cuvettes at 20 °C. The bandwidth used was 1 nm with a scan time per point of 1 s. For each sample, two repeat scans were averaged and baseline-corrected by subtraction of the blank buffer. CD spectra deconvolution was performed using a CDNN (version 2.1) software tool (Applied Photophysics, UK) in the 195–260 nm spectral region because of its accuracy over the entire spectral region examined (i.e. with a total percentage sum closest to 100%).

### Substrate synthesis

Tetra(glycerol)orthosilicate (TGS) was synthesised as described previously^13^. It should be noted that TGS can not be accurately characterized in a standard way. We studied the 1H NMR spectra of the obtained substance immediately after the synthesis and after several variants of purification and found that it is an identical mixture in all cases. The NMR spectra of this mixtures characterized by two broad groups of multiplets in area of 4.3–4.8 ppm (corresponds to OH groups) and in area of 3.2–4.2 ppm (corresponds to CH and CH_2_ groups) whose shape were more or less identical from one sample to another (Supplementary Fig. S3). Probably, it represents a certain equilibrium mixture of different silicates of glycerol substituted by 1st and/or 2nd alcohol groups, containing different linear and cyclic, as well as monomeric and oligomeric derivatives. Formation of this equilibrium mixture occurs quickly enough, that does not allow us to purify the individual tetraglyceride, but the consistency of its composition was confirmed by the reproducibility of the results of the repeatedly performed synthesis and the identity of the obtained spectra. Thus, the name “TGS” should be treated not as the name of an individual compound, but as the ratio of the residues of glycerol to the silicon atom in a complex mixture.

As TGS was used without purification and the presence of free glycerol in obtained mixtures was revealed by NMR (Supplementary Fig. S3), possible influence of glycerol on silica-condensing activity was tested. These control experiments showed that silica polymerizing activity of LoSilA1 was unaffected by as high as 10-fold excess of glycerol (0.1% TGS, 1% glycerol) (not shown).

### Silica-condensing activity measurement

To evaluate silica-condensing activity, 1 ml of the 0.1% TGS solution was added to the 600 µl of the protein solution (0.06 mg/ml). After 1 h of incubation in the desired conditions samples were centrifuged, washed 3 times with 96% ethanol, air dried and dissolved in 200 µl 2M NaOH. The amount of the polymerized silica was determined by the colorimetric molybdate assay as described previously^13^. In spite of efforts to standardize experimental conditions, we observed rather strong difference in the absolute amount of the polymerized silica in different experiments. The probable reason for this is a poor stability of the purified proteins. To solve this problem, the wild-type protein, its mutants, and reference silicatein LoSilA1 were expressed, purified and measured in parallel in every experiment (4–5 independent experiments for all sets of mutants). Normalized data obtained in such a way were highly reproducible. Also, there was a negative control of spontaneous silica precipitation without any proteins in every experiment. Spontaneous TGS polymerization was at least 20-fold lower than in any sample with silicatein/cathepsin L. This background polymerization value was subtracted from every sample TGS + protein.

### SEM-XRF

Protein solution (198 µl, 0.1 mg/ml) was mixed with 2 µl of 10% TGS and incubated for 1 h. Then, the samples were centrifuged (12,000 g) for 10 min, washed 1 time with 96% ethanol, incubated in 6 M guanidine hydroxide for 1 h, washed 1 time with water and 2 times with 70% ethanol, air dried, resuspended in 200 µl of deionized water and vortexed. Sample solution (5 µl) was deposited onto freshly cleaved highly oriented pyrolytic graphite (ZYB quality, NT-MDT, Russia) and dried in a vacuum chamber.

Energy-dispersive X-ray spectroscopy (EDS) microanalysis was performed by SEM (Zeiss MERLIN, emission electron microscope with ESEM capability) as described previously^13^.

## Electronic supplementary material

Supplementary Information

## Data Availability

The datasets generated during the current study are available from the corresponding author on reasonable request.
