# Supplementary material for: Efficient silica synthesis from tetra(glycerol)orthosilicate with cathepsin- and silicatein-like proteins
Source: Sci Rep. 2018 Nov 13;8:16759. doi: 10.1038/s41598-018-34965-9 (PMC6233156; doi:10.1038/s41598-018-34965-9)
Supplement: Supplementary file 1 — Supplementary Information [file 41598_2018_34965_MOESM1_ESM.pdf]

Efficient silica synthesis from tetra(glycerol)orthosilicate with cathepsin- and silicatein-like proteins

Natalia V. Povarova, Nikolay A. Barinov, Mikhail S. Baranov, Nadezhda M. Markina, Anna M. Varizhuk, Galina E. Pozmogova, Dmitry V. Klinov, Valery B. Kozhemyako & Konstantin A. Lukyanov

Supplementary Information

Supplementary Figure S1	Scanning electron microscopy analysis of silica-polymerizing activity of CTSL and its mutants
Supplementary Figure S2	Silicatein-cathepsin chimera structure
Supplementary Figure S3	1H NMR spectrum of TGS

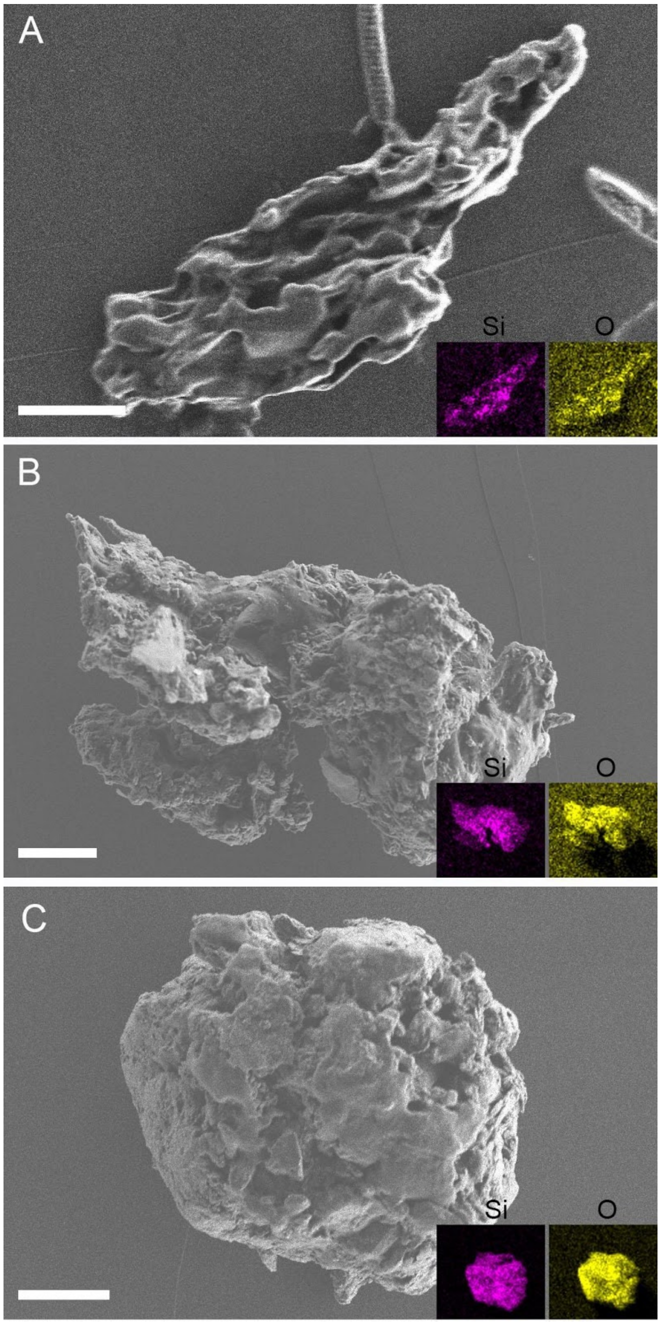

Supplementary Figure S1. Scanning electron microscopy analysis of silica-polymerizing activity of CTSL and its mutants. Silica particles were formed with TGS in Tris-HCl buffer by CTSL (**A**), CTSL-C25A (**B**), and CTSL-H163A (**C**). Scale bars 2 μm. Corresponding XRF maps of Si (magenta) and O (yellow) distribution are in the insets.

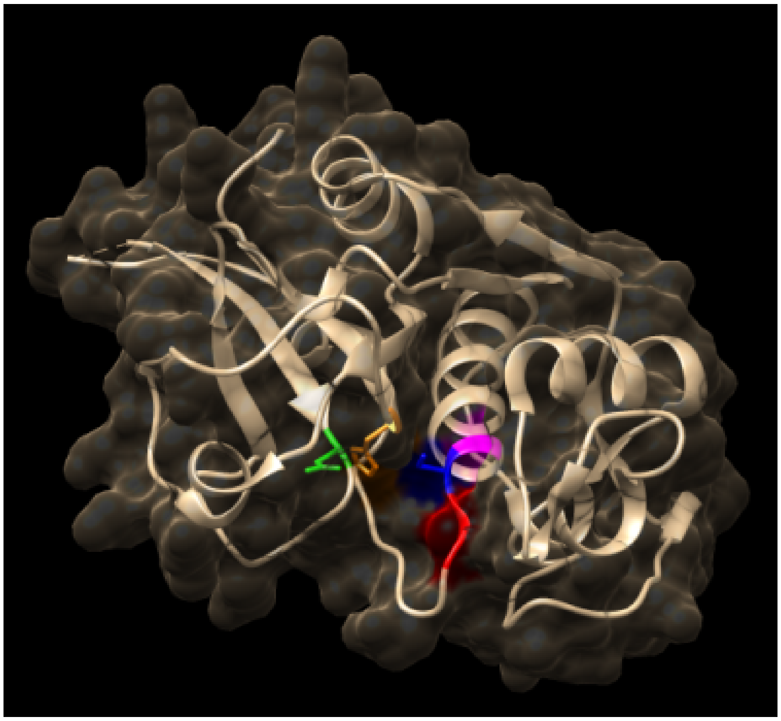

Supplementary Figure S2. Silicatein-cathepsin chimera structure. Shown is secondary structure and surface (PDB ID 2VHS); with marked location of the mutated residues: Gly23 and Ala24 – red, Ser25 – blue, Tyr26 – magenta, His163 – orange, Asn187 – green. The picture was produced by UCSF Chimera: Pettersen, E. F. et al. UCSF Chimera--a visualization system for exploratory research and analysis. J. Comput. Chem. 25, 1605–1612 (2004).

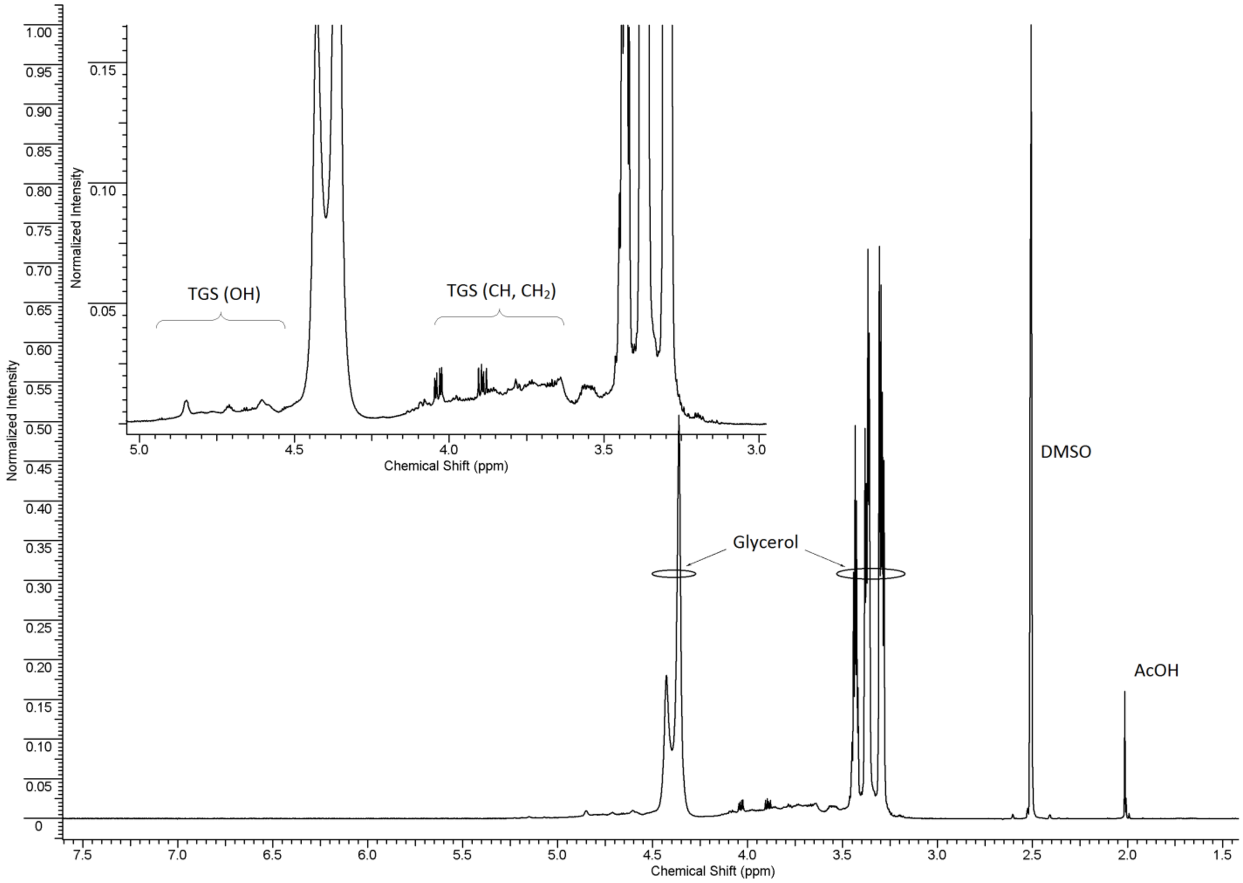

Supplementary Figure S3. Representative 1H NMR spectrum of TGS sample.
